# Plasmodium genus- and species-specific real-time PCR using SYBR dye decreases laboratory time without impairing the sensitivity or specificity compared to conventional PCR

**DOI:** 10.1186/1475-2875-13-S1-P39

**Published:** 2014-09-22

**Authors:** Christel Gill Haanshuus, Stein Christian Mohn

**Affiliations:** 1 Department of Medicine, National Centre for Tropical Infectious Diseases, Haukeland University Hospital, Bergen, Norway

## Background

A newly published genus-specific PCR targeting mitochondrial genome was highly sensitive and specific (Haanshuus *et al.* 2013). The aim of the study was to decrease the laboratory time without impairing sensitivity or specificity, by converting the conventional PCR to real-time PCR using SYBR dye. A second aim was to include species-specific assays to be optionally run together with the genus-specific real-time assay.

## Materials and methods

Using a well-defined patient material, previously described (Haanshuus *et. al* 2013), a real-time PCR using SYBR dye was developed. The protocol included genus-specific mitochondrial primers (Polley *et al.* 2010), as well as 18S species-specific primers (Padley *et al.* 2003, Haanshuus *et al.* 2013), though with separate master mixes for each target. As opposed to the conventional PCR the assays could be optionally run together, as the master mixes were optimized to fit the same standard cycling parameters. The material consisted of 26 confirmed positives and 82 negative ones. In addition a ten-folded dilution series of a standardized reference sample was included. Limit of detection was determined. All the positives and the dilution series were analyzed in duplicates. Threshold cycle (Ct) cut-off value was set at 35.

## Results

The mitochondrial genus-specific real-time assay detected 24 of the 26 positives in both duplicates (17 *P. falciparum*, five *P. vivax*, one *P. ovale*, and one mixed infection with *P. falciparum* and *P. malariae*). A submicroscopic *P. malariae* was not positive in any of the duplicates, while a *P. falciparum* sample was positive in one out of two. The latter sample had previously not been detected by any 18S PCR assays (Haanshuus *et al.* 2013). The limit of detection was 0.2-2 parasite/μl. (The conventional genus-specific assay had a sensitivity of 0.5 p/μl). The specificity was 100%. The 18S species-specific assays detected as well 24 out of the 26 positives in both duplicates, with 100% specificity (no cross-binding). The submicroscopic *P. malariae* and the mentioned *P. falciparum* were not detected in any of the duplicates. The sensitivity was 100% screening the negatives.

## Conclusions

The real-time assays showed similar high sensitivities and specificities as its conventional counterparts, though less time-consuming and more practical and user-friendly both for research purposes and clinical diagnostics. The sensitive and efficient genus-specific assay is ideal for screening large sample sizes; however, the species-specific assays can be optionally run simultaneously which is an advantage especially in clinical diagnostics.

